# Melatonin Ameliorates the Toxicity Induced by Deoxynivalenol in Murine Ovary Granulosa Cells by Antioxidative and Anti-Inflammatory Effects

**DOI:** 10.3390/antiox10071045

**Published:** 2021-06-29

**Authors:** Hairui Fan, Shiqin Wang, Haifei Wang, Mingan Sun, Shenglong Wu, Wenbin Bao

**Affiliations:** 1 Key Laboratory for Animal Genetics, Breeding, Reproduction and Molecular Design, College of Animal Science and Technology, Yangzhou University, Yangzhou 225009, China; DX120180095@yzu.edu.cn (H.F.); MX120180624@yzu.edu.cn (S.W.); hyfiwang@yzu.edu.cn (H.W.); slwu@yzu.edu.cn (S.W.); 2 College of Veterinary Medicine, Yangzhou University, Yangzhou 225009, China; mingansun@yzu.edu.cn; 3 Joint International Research Laboratory of Agriculture & Agri-Product Safety, the Ministry of Education of China, Yangzhou University, Yangzhou 225009, China

**Keywords:** melatonin, ovary granulosa cells, deoxynivalenol, apoptosis, antioxidation, anti inflammatory

## Abstract

Melatonin is an important endogenous hormone that shows antioxidant functions and pleiotropic effects, playing a crucial role in animal reproduction. Ovary granulosa cells (GCs) surround the oocyte, which play an important role in regulating oocytes development. Deoxynivalenol (DON) is a common fusarium mycotoxin contaminant of feedstuff and food, posing a serious threat to human and animal reproductive systems. Herein, murine ovary GCs were studied as a reproduction cell model, aimed to assess the protective effect of melatonin on DON-induced toxicity in murine ovary GCs. The results showed that DON adversely affected the viability and growth of murine ovary GCs and increased the apoptosis rate, while melatonin administration ameliorated these toxic effects. We further reveal that DON exposure increased the intracellular reactive oxygen species level, reduced the mitochondrial membrane potential and ATP, and upregulated Tnfα (tumor necrosis factor α), Il6 (interleukin 6), and Il1β (interleukin 1 β) gene expression. Moreover, DON exposure downregulated reproductive hormone gene expression and significantly increased nuclear factor kappa B (p65) activation and mitogen-activated protein kinase phosphorylation. Melatonin treatment attenuated all these effects, suggesting that melatonin protects GCs from the adverse effects of DON by ameliorating oxidative stress, mitochondrial dysfunction, and inflammation. Overall, these results reveal the mechanisms of DON and melatonin in GCs and provide a theoretical basis for melatonin as a drug to improve mycotoxin contamination.

## 1. Introduction

**Figure 1:**
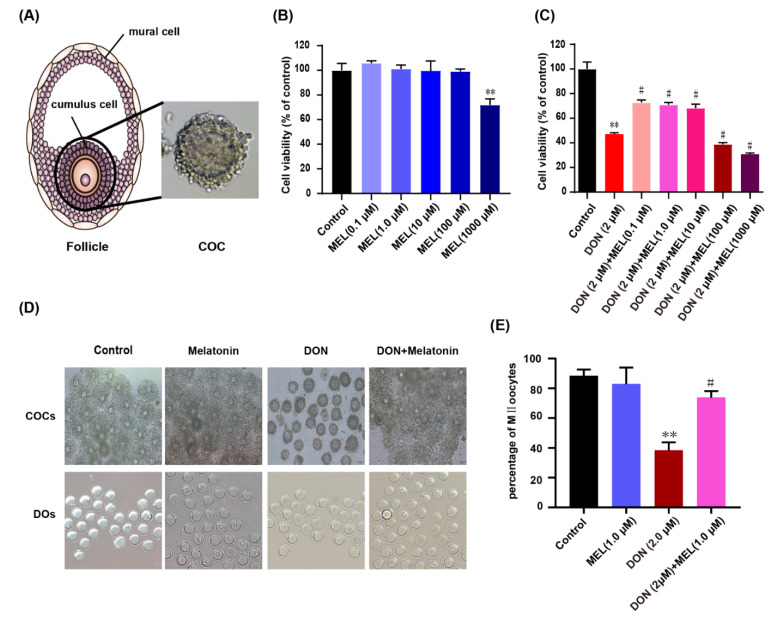
The effect of melatonin and DON on murine ovary GCs and oocytes. (**A**) The mode pattern of mammalian follicle and cumulus–oocyte complex (COC) (Magnification: 400×). (**B**) The effects of gradient concentration of melatonin on GCs Cell viability for 24 h. (**C**) The effects of combined treatment melatonin and DON on GCs cell viability for 24 h. Cell viability was determined using the CCK-8 assay. (**D**) Oocyte morphologies in the control, Melatonin-exposed, DON-exposed, and melatonin + DON groups. Scale bar: 100 µm. (**E**) The effects of melatonin on the rate of polar body extrusion in DON-exposed oocytes. The data are expressed as the means ± SEM. ** *p* < 0.05 compared with the control; # *p* < 0.05 compared with the DON group. The results showed that appropriate melatonin significantly inhibited DON-induced decreases in cell viability. MEL: melatonin.

Many follicles at different stages of maturation present in the mammalian ovary, and each follicle contains an oocyte surrounded by granulosa cells (GCs), maintaining the structure of cumulus–oocyte complex (COC) (Figure 1A) for a period of time. The specialized structure of paracrine and junctional interactions between GCs and oocyte plays a key role in carefully regulating oocytes development [1], including providing substate to the growing oocytes [2], and regulating follicular selection and atresia [3]. Particularly, steroid hormones synthesized by GCs play an important role in follicles development [4,5].

Melatonin (*N*-acetyl-5-methoxytryptamine) is a major hormone secreted by the pineal gland, which is involved in biological rhythm regulation and seasonal reproductive activity in mammals [6]. Recent research reported that melatonin can be produced by many other tissues, including the gastrointestinal tract [7], immune cells [8], and reproductive system cells [9,10]. Furthermore, it has been widely used in a variety of products to improve the quality of human sleep. Meanwhile, melatonin is also widely recognized as an antioxidant and free radical scavenger [11,12], and has been shown recently to have pleiotropic effects [6,13]. Melatonin and its metabolites have been reported to activate a variety of antioxidant enzymes, and are involved in antioxidative stress and the inhibition of cell apoptosis [14]. In the reproductive system, accumulating research has proven that melatonin plays an important role via regulation of antioxidative stress, especially the steroidogenesis capacity in the development of ovarian GCs [15], oocytes [11], pronuclear embryo, embryo implantation, and litter size [16]. Melatonin supplementation in pig ovary GCs in vitro contributed to the synthesis of estradiol, helped to regulate the mRNA expression of reproductive hormone-related genes [15], and upregulated the mRNA expression of oocyte maturation associated genes, such as *GDF9* (growth differentiation factor 9) and *DNMT1A* (DNA methyltransferase 1) [17]. Moreover, melatonin protected porcine oocytes from heat stress [14] and Di-2-ethylhexyl phthalate (MEHP) (plasticizer) [18] exposure-induced meiosis defects via its antioxidant properties. In addition, melatonin could effectively eliminate free radicals in oocytes during ovulation [10]. Deoxynivalenol (DON) is the main mycotoxin secreted by the *Fusarium* fungus, which frequently contaminates grain and feed [19,20]. Generally, adulteration with DON is accompanied by contamination with other mycotoxins, such as 3-acetyl-DON, 15-acetyl-DON, and DON-glucoside [21], which possess the same main functional groups as DON. A previous study demonstrated that even though extrusion cooking can effectively promote the degradation of DON, it cannot completely eliminate DON [22]. The high chemical stability of DON allows it to be easily absorbed via feed intake into the gastrointestinal tract [23], and then enter various other organs [24]. Consequently, DON exerts severe detrimental effects, causing a series of pathophysiological symptoms in humans and animals, including nausea, diarrhea, vomiting, leukocytosis, hemorrhage, and even death [25]. Therefore, DON contamination poses a serious threat to human health and animal husbandry. Animal experiments have shown that acute exposure to high doses of DON induces oxidative stress [26], leukocytosis, gastrointestinal bleeding, circulatory failure, and eventually death. Meanwhile, exposure to long-term medium dose food intake resulted in endocrine changes, which can cause body weight decrease, immune dysfunction, and affect development and animal fertility [27]. In the reproductive system, DON inhibits the proliferation of ovarian granulosa cells (GCs) and endometrial cells [11,28], and alters the synthesis of testosterone, progesterone, and estradiol in ovarian GCs [29,30], which adversely affects oocyte maturation and embryo development [11,31]. Moreover, the specialized structure of ovarian GCs with oocytes has a crucial effect on the development of follicles, oocytes [1,15], subsequent zygote quality, and embryonic development. However, the specific mechanisms of the toxic effects of DON on murine ovary GCs remain unclear, and it is unclear whether melatonin could rescue the toxic effects of DON exposure on mouse ovary GCs.

In the present study, we aimed to investigate the toxic effects and mechanisms of DON exposure on murine ovary GCs, and to explore whether melatonin administration could protect against the detriments induced by DON.

## 2. Materials and Methods

### 2.1. Chemicals and Reagents

DON (D0156; 5 mg) and melatonin (M5250; 250 mg) were purchased form Sigma-Aldrich (St. Louis, MO, USA). Fetal bovine serum and Dulbecco’s modified Eagle’s medium (DMEM)-F12 were obtained from Gibco (Invitrogen Corporation, Carlsbad, CA, USA). Anti-BCL2 associated X, apoptosis regulator (Bax; ET1603-34), anti-BCL2 apoptosis regulator (Bcl-2; ET1603-11), anti-caspase 3 (ER30804), anti-cleaved caspase 3 (ET1608-64), anti-caspase 9 (ET1603-27), anti-follicle stimulating hormone receptor (FSHR; ER1909-08), and anti-rabbit immunoglobulin G (IgG)-horseradish peroxidase (HRP; HA1031) antibodies were purchased form Hangzhou HuaAn Biotechnology (Hangzhou, China). Anti-androgen receptor (AR; ab273500), anti-extracellular regulated kinase 1 (ERK1; ab109282), anti-phospho-ERK (ab201015), anti-p65 (ab32536), anti-phospho-p65 (ab76302), anti-p38 (ab170099), anti-phospho-P38 (ab178867), anti-IκB (ab32518), anti-phospho-IKB (ab133462), anti-JNK (ab179461), and anti-phospho-JNK (ab124956) antibodies were obtained from Abcam Ltd. (Cambridge, UK). Anti-heat shock protein 90 (HSP90; 60318) and anti-GAPDH (10494-1-AP) antibodies were obtained from Proteintech Ltd. (Proteintech, Rosemont, IL, USA).

### 2.2. Isolation and Culture of Ovarian GCs and Oocytes

Four-week-old and 18–20 g BW (body weight) Institute of Cancer Research (ICR) female mice were housed in an appropriate temperature-controlled room with a 12-h light-dark cycle (on at 8:00 a.m., off at 20:00 p.m.), and fed with a regular diet and water. Total 120 ICR female mice were used in the experiments. Mice were injected with 6 IU (international unit) pregnant mare serum gonadotrophin (Ningbo Hormone Products Co., Ltd., Ningbo, China), and then at 44–48 h later, their ovaries were collected.

Ovarian GCs were collected from the isolated ovaries and washed three times in serum-free DMEM/F12. The GCs were dispersed with DMEM/F12 supplemented with 10% FBS, and seeded at a density of 5 × 10^4^ cells/mL in 96-well and 6-well plates. The cells were incubated in 5% CO_2_ at 37 °C for 24 h before further treatment, and the culture medium was refreshed every 24 h. The cumulus–oocyte complexs (COCs) were collected from these ovaries, after being washed three times in M2 medium, and then cultured in IVM medium (M2115, Nanjing Aibei, Nanjing, China) under paraffin oil at 37 °C in a 5% CO_2_. After culture for 17 h, the denuded oocytes (DOs) were collected after removal of GCs.

### 2.3. DON and Melatonin Treatment

DON was dissolved in ethanol (the original concentration was 10 mM) and diluted in culture medium to a final concentration of 2 μM. We previous examined the cell viability of GCs treated with different concentrations of DON (0.1, 1, 2, and 3 μM), which revealed that cell viability decreased gradually with increased concentration of DON. Cell viability can be decreased to ~50% when treated with 2 μM DON, and further increasing of the concentration to 3 μM does not remarkably decrease the cell viability. In addition, a previous study also applied DON at a concentration of 2 μM to treat murine ovary oocytes, and found that DON adversely affected mouse oocyte maturation and early embryo cleavage [11]. Melatonin was dissolved in ethanol (1 M) and diluted in culture medium to 2 μM DON-contaminated culture medium to final concentrations of 0.1, 1.0, 10, 100, and 1000 μM. The final concentration of the solvent was less than 0.1% in the culture medium. Cells and COCs treated with the control (an equal volume of solvent), DON (2 μM), melatonin (1.0 μM), and DON (2 μM) + melatonin (1.0 μM) were analyzed in subsequent trials.

### 2.4. Assessment of Cell Viability

After the GCs grew to 70% confluency in 96-well plates, they were treated with DON (2 μM), melatonin (0.1, 1.0, 10, 100, and 1000 μM), and DON (2 μM) + melatonin (0.1, 1.0, 10, 100, and 1000 μM) for 24 h. Cell viability was assessed using a Cell Counting Kit-8 (Dojindo Laboratories, Kumamoto, Tokyo, Japan) according to the manufacturer’s protocol. The optical density (OD) measurement was carried out at a wavelength of 450 nm on a Tecan Infinite 200 microplate reader (Sunrise, Tecan, Switzerland).

### 2.5. Analysis of Apoptosis Using Flow Cytometry

After the GCs grew to 70% confluency in 6-well plates, they were treated with DON (2 μM), melatonin (1.0 μM), and DON (2 μM) + melatonin (1.0 μM) for 24 h, and the control group was cultured in medium with an equal volume of solvent. GCs were collected to checked apoptosis via Annexin V-fluorescein isothiocyanate (FITC)/propidium iodide (PI) Apoptosis Detection Kit (Solarbio, Beijing, China). Briefly, collected GCs were washed in ice cold phosphate-buffered saline (PBS), stained with Annexin V-FITC and propidium iodide (PI) in order, subjected to flow cytometry, and then analyzed using CytExpert 2.3 (Beckman Coulter, Brea, CA, USA); each measurement was performed three times using at least 10,000 cells.

### 2.6. Measurement of Reactive Oxygen Species (ROS) Using Flow Cytometry

ROS in GCs were monitored using flow cytometry using a Reactive Oxygen Species Assay Kit (Solarbio, Beijing, China). After treatment with DON (2 μM), Melatonin (1.0 μM) and DON (2 μM) + Melatonin (1.0 μM) for 24 h, the cells were collected, washed in pre-cooled PBS twice, and then incubated with 10 μM dichloro-dihydro-fluorescein diacetate (DCFH-DA) in culture medium at 37 °C for 30 min. The cells were collected, washed in PBS three times, resuspended in PBS, and then the intracellular ROS were measured using a flow cytometer (Beckman Coulter, Brea, CA, USA). Each measurement was performed for at least 20,000 cells.

### 2.7. Measurement of the Mitochondrial Membrane Potential (ΔΨm)

The Mitochondrial Membrane Potential (Δ*Ψm*) was determined quantitatively by using a JC-1 dye Detection Kit (Beyotime, Jiangsu, China) by flow cytometry. JC-1 dye is an ideal fluorescent probe to detect Δ*Ψm*, and is suitable for cells, tissues, and purified mitochondria. When the Δ*Ψm* is high, JC-1 accumulates in the mitochondrial matrix to form JC-1 aggregates, presenting as red fluorescence; however, when the Δ*Ψm* is lower, JC-1 dye accumulates in the cytoplasm as monomers, presenting as green fluorescence. Thus, it is easy to detect a decrease in the Δ*Ψm* by observing the transition of JC-1from red fluorescence to green fluorescence. Therefore, the ratio of the red to green fluorescence intensity, as detected using flow cytometry, represents the quantitative Δ*Ψm* in each group. Briefly, after the GCs were treated with DON (2 μM), melatonin (1.0 μM) and DON (2 μM) + melatonin (1.0 μM) for 24 h, they were collected and stained by JC-1 dye at 37 °C for 20 min. The cells were then washed three times and resuspended in 0.5 mL of the per-cooled buffer solution, and the fluorescence was quantitatively determined using CytExpert 2.3 (Beckman Coulter, Brea, CA, USA).

### 2.8. Determination of ATP Content

The ATP content of GCs was determined using an Enhanced ATP Assay Kit (Beyotime, Shanghai, China). The treated GCs from the four groups were collected and counted using cell-count boards, and then diluted to the same number of cells (1 × 10^7^) in each group. The ATP content was then determined following the manufacturer’s instructions. Briefly, the collected cells were lysed with lysis buffer on ice for 20 min, and then centrifuged at 12,000× *g* for 5 min at 4 °C, and the supernatant was retained. Meanwhile, the ATP working solution was added at 100 μL/well and kept at room temperature for 5 min. Then, ATP standard solutions diluted at different concentration and the different supernatant samples were added to the 96-well plate (20 μL/well), mixed with ATP working solution quickly, and subsequently measured using a BioTek Synergy 2 Multimode Microplate Reader (BioTek, Winooski, VT, USA). The ATP content was quantified according to the established standard curve.

### 2.9. RNA Extraction and Quantitative Real-Time Reverse Transcription PCR (qRT-PCR)

The GCs were collected after treatment with DON (2 μM), Melatonin (1.0 μM) and DON (2 μM) + Melatonin (1.0 μM) for 24 h in a 6-well plate. Then, total RNA was extracted using a YEASEN Total RNA Extraction Reagent (YEASEN, Shanghai, China), and the RNA quantity was measured using 260/280 UV spectrophotometry. Equal quantities of RNA were reverse transcribed into cDNA using an HiScript^®^ Q RT SuperMix for qRT-PCR (+gDNA wiper) kit (Vazyme, Nanjing, China). Then, quantitative real-time PCR (qPCR) reactions were carried out using the cDNA as the template and the AceQ^®^ qPCR SYBR^®^ Green Master Mix (Vazyme, Vazyme Biotech, Nanjing, China) in an ABI StepONEPlus Real-Time PCR System (Applied Biosystems, Foster City, CA, USA), using the following protocol: 95 °C for 5 min; followed by 40 cycles of 95 °C for 10 s, 60 °C for 30 s. All the specific primers used for qPCR are listed in Table S1. The mouse *Gapdh* mRNA was used as an internal control. Relative gene expressive was calculated using the 2^−ΔΔCt^ method [32].

### 2.10. Western Blotting Analysis

The treated GCs were collected, washed with pre-cooled PBS, and then lysed with radioimmunoprecipitation assay (RIPA) buffer supplemented with protease and phosphatase inhibitors for 10 min on ice, and centrifuged at 13,000× *g* for 10 min at 4 °C. The protein concentration was determined using a bicinchoninic acid (BCA) protein assay kit (Biosharp, Beijing, China). Equal amounts of protein (20 μg) were loaded in each well and separated on an 8% or 10% sodium dodecyl sulphate–polyacrylamide gel, and then transferred onto polyvinylidene difluoride (PVDF) membranes (Bio-Rad, Hercules, CA, USA). The membranes were washed thrice with Tris-Buffered Saline Tween-20 (TBST) and then blocked with 5% nonfat dry milk in TBST at room temperature for 2 h with gentle agitation. Subsequently, the blocked membranes were incubated with primary antibodies (diluted 1:1000) at 4 °C overnight. The membranes were washed thrice with TBST (10 min each), and then incubated with anti-rabbit IgG-HRP secondary antibody (1:5000) at room temperature for 1 h with gentle agitation. The membranes were washed thrice with TBST (10 min each), and then incubated with Luminol/Enhancer Reagent (New Cell & Molecular Biotech, Suzhou, China) and exposed to the FluorChem FC3 system (Protein-Simple, San Jose, CA, USA). Finally, the relative integrated density of each protein band was digitized using the FluorChem FC3 system. GAPDH or HSP90 was selected as a control for equal protein loading.

### 2.11. Data Analysis and Statistics

The results are presented as means ± SD with at least three samples, or the mean ± SEMs using at least three biological replicates. Statistical analyzes were performed and the figures were generated using GraphPad Prism software (version 8.0, GraphPad, Inc, San Diego, CA, USA). Statistical comparisons used an unpaired *t*-test. *p* value < 0.05 was considered statistically significant.

## 3. Results

### 3.1. Effect of Melatonin on DON-Induced Cell Viability

The cell viability of ovarian GCs treated with DON, melatonin, and DON + melatonin for 24 h was determined using the CCK-8 assay. When ovary GCs were treated with melatonin separately at different concentrations (0.1, 1.0, 10, 100 and 1000 μM), only melatonin of 1000 μM decreased cell viability significantly (*p* < 0.05) (Figure 1B). We treated ovary GCs with DON (2 μM) and DON (2 μM) + melatonin (0.1, 1.0, 10, 100 and 1000 μM). The result showed that DON (2 μM) decreased cell viability significantly (*p <* 0.01), whereas co-treatment with low concentrations of melatonin (0.1, 1.0, and 10 μM) attenuated the cytotoxicity of DON significantly (Figure 1C). The cell confluency of the DON (2 μM) + melatonin (1.0 μM) group was significantly higher than that in the DON-treat group, and the cell morphology also seemed normal (Figure S1). These results revealed that an appropriate amount of melatonin could rescue the cytotoxicity induced by DON in ovarian GCs.

### 3.2. Melatonin Protects against Meiotic Maturation Defects in DON Exposure Mouse Oocytes

To further verify the toxic effects of DON and protective effect of melatonin on ovary GCs. The IVM (in vitro maturation) of oocytes was executed with different treatment (control, DON (2 μM), melatonin (1.0 μM) and DON (2 μM) + melatonin (1.0 μM)). Interestingly, we found that DON significantly inhibited the expansion of cumulus cells of COCs during the process of IVM compared to the control and melatonin group, and melatonin co-administration groups resorted significantly to the expansion of cumulus cells of COCs (Figure 1D). Meanwhile, the rate of polar body extrusion was recorded. We found that DON exposure inhibited significantly mouse oocyte maturation compared to the control and melatonin groups (38.31 ± 5.48%, *n* = 125 vs. 88.26 ± 4.39%, *n* = 400 and 82.81 ± 11.16%, *n* = 155, *p* < 0.01), and melatonin co-administration significantly increased the rate of polar body extrusion in DON exposure oocytes (73.7 ± 4.39%, *n* = 113 vs. 38.31 ± 5.48%, *n* = 125; *p* < 0.01) (Figure 1E). These results indicated that melatonin protect cumulus cells and against meiotic maturation defects induced by DON during the process of IVM.

### 3.3. Melatonin Decreases DON-Induced Murine GCs Apoptosis

**Figure 2:**
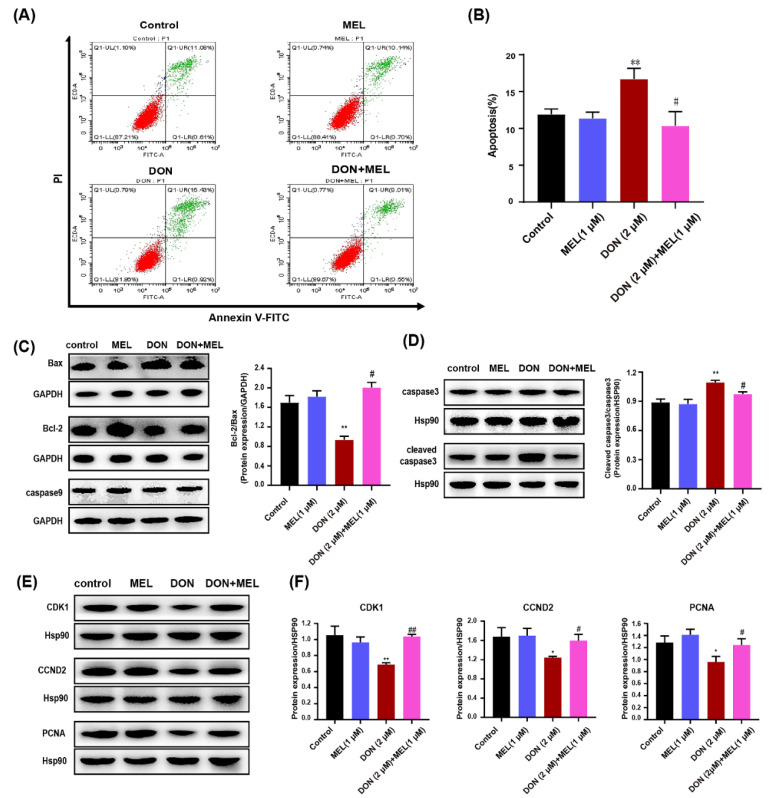
Melatonin treatment ameliorates DON-induced mouse ovary GC apoptosis. (**A**) Apoptosis of GCs was evaluated by measurement of Annexin V using flow cytometry. Apoptotic cells were Annexin V-positive and PI (propidium iodide)-negative. (**B**) The figure shows representative staining, and the numbers in the quadrants indicate the percentage of cells within the respective subpopulations. (**C**,**D**) Western blotting analysis of the changes in the protein levels of related apoptosis factors (Bax, Bcl-2, cleaved caspase-3, caspase-3, caspase9). (**E**,**F**) Western blotting analysis of the changes in the protein level of functional genes linked to proliferation (PCNA, CDK1, and CCND2). Each bar represents the mean ± SD from three different experiments, *n* = 3. * *p* < 0.05 and ** *p* < 0.01 compared with the control and melatonin (MEL) groups; # *p* < 0.05, ## *p* < 0.01 compared with the DON group. MEL: melatonin.

To further determine the harmful effects of DON and the protect effect of melatonin on DON-induced cell cytotoxicity in ovary GCs, we tested the apoptotic rate of GCs pretreated with DON (2 μM), melatonin (1.0 μM), and DON (2 μM) + melatonin (1.0 μM) for 24 h. DON significantly increased the ratio of apoptotic cells compared with that of the control group (control: 11.93 ± 0.71%; DON group: 16.73 ± 1.43%), while melatonin had no effect on the apoptotic rate (11.38 ± 0.83%) (Figure 2A,B). Interestingly, treatment with DON + melatonin reduced the rate of cell apoptosis cells (10.38 ± 1.90%) to a level similar to the control group (Figure 2A,B). We further tested the level of several related apoptosis factors (i.e., Bcl-2, Bax, caspase 3, cleaved caspase 3 and caspase 9), and found that DON significantly induced ovarian GC apoptosis as indicated by a decrease in the Bcl-2/Bax ratio and an increase in the cleaved caspase 3/caspase 3 ratio (Figure 2C,D). Co-treatment with melatonin ameliorated these effects compared with those in the DON group, i.e., it restores the levels of Bcl-2, Bax, cleaved caspase 3 as well as Bcl-2/Bax, and cleaved caspase 3/caspase 3 ratio to a degree similar to control (Figure 2C,D). Moreover, we also measured the protein level of functional genes linked to proliferation, such as PCNA, CDK1, and CCND2. The result showed that DON exposure exhibited reduced levels of CDK1, CCND2, and PCNA, and co-treatment with melatonin ameliorated these effects compared with those in the DON group (Figure 2E,F). Taken together, our results demonstrated that melatonin treatment ameliorated DON-induced apoptosis and facilitated the proliferation of murine ovary GCs.

### 3.4. Melatonin Decreases ROS Production in DON-Exposed Ovary GCs

**Figure 3:**
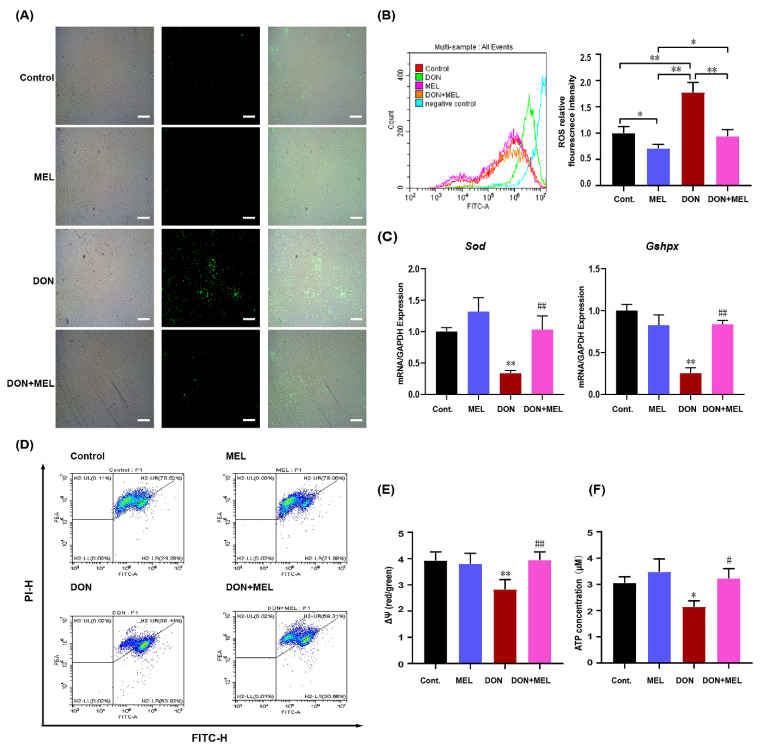
Effects of melatonin on ROS levels and mitochondrial function of murine ovary GCs upon DON exposure. (**A**) Representative images of ROS levels in the control, melatonin (MEL)-exposed, DON-exposed, and MEL + DON-treated mouse ovary GCs. Scale bar, 300 μm; (**B**) Fluorescence intensities of ROS in the mouse ovary GCs of the different groups; (**C**), qRT-PCR analysis of the relative expression of *Sod* and *Gshpx* mRNA levels. (**D**) Mitochondrial membrane potential levels of the different groups. (**E**) The ratio of the red over green fluorescence intensity by flow cytometry represents the quantitative Δ*Ψm* in each group. (**F**) ATP content of the different groups. All values are expressed as the means ± SD (*n* = 3), * *p* < 0.05 and ** *p* < 0.01 versus the control group; # *p* < 0.05 and ## *p* < 0.01 versus the DON group. MEL: melatonin.

DON can induce oxidative stress in many cell types to impair cellular function [11]. Therefore, we examined the ROS levels in the GCs after DON exposure for 24 h. The results showed that DON induced oxidative stress in ovary GCs. The ROS signal was significantly increased in the DON exposure group compared with that in the control group, while the ROS levels in the melatonin and DON + melatonin groups were similar to those in the control group (Figure 3A). Moreover, ROS relative fluorescence intensity analysis indicated that the addition of melatonin decreased the ROS production level significantly compared with that in the DON group (control: 1.00 ± 0.07; melatonin: 0.71 ± 0.05; DON: 1.77 ± 0.11; melatonin + DON: 0.94 ± 0.06) (Figure 3B). To further determine the antioxidative effect of MEL in ovary GCs upon DON exposure, we tested the mRNA expression of *Sod* and *Gshpx,* which encode the main antioxidant enzymes superoxide dismutase (SOD) and glutathione peroxidase (GPX). Compared with the control group, GCs treated with DON exhibited decreased levels of *Sod* mRNA expression (0.34 ± 0.04 vs. 1.00 ± 0.06, *p <* 0.01), while its expression in the melatonin + DON group was upregulated significantly (1.03 ± 0.22, *p <* 0.01), which was not different from its expression in the melatonin (melatonin: 1.32 ± 0.22) and control groups (Figure 3C). The expression trend of *Gshpx* was similar to that of *So*d (DON: Control: Melatonin, 0.25 ± 0.06 vs. 1.00 ± 0.07 vs. 0.83 ± 0.12, *p <* 0.01; melatonin + DON: DON, 0.84 ± 0.06 vs. 0.25 ± 0.06, *p <* 0.01) (Figure 3D). These results illustrated that melatonin alleviated oxidative stress in murine ovary GCs induced by DON.

### 3.5. Melatonin Ameliorates DON-Induced Mitochondrial Dysfunction

To further verify the protective effect of melatonin on mitochondria in murine ovary GCs upon DON exposure, the Δ*Ψm* and ATP content in GCs were measured after different treatments. The result showed that the addition of DON increased the level green fluorescence (Figure 3D, DON) compared with the control group (Figure 3D, control) and melatonin group (Figure 3D, MEL), while the addition of melatonin decreased the level green fluorescence (Figure 3D, DON + melatonin) compared with the DON group. Meanwhile, we quantified the alteration in Δ*Ψm* using the ratio of red fluorescence intensity to green fluorescence intensity (Figure 3E). After DON treatment, the ratio decreased significantly compared with that of the control group (2.83 ± 0.17 vs. 3.92 ± 0.19, *p <* 0.01), while co-treatment with melatonin and DON mitigated the effect significantly (3.91 ± 0.18 vs. 2.83 ± 0.17, *p <* 0.01). Moreover, DON decreased the ATP content significantly in ovary GCs compared with that in the control and melatonin groups (2.14 ± 0.13 vs. 3.05 ± 0.14 vs. 3.48 ± 0.28), while these effects were mitigated by co-treatment with melatonin and DON (3.23 ± 0.21 vs. 2.14 ± 0.13, *p <* 0.05) (Figure 3F). These results showed that melatonin alleviated the mitochondrial dysfunction in ovarian GCs induced by DON.

### 3.6. Melatonin Inhibits DON-Induced Inflammatory Cytokine Gene Expression

**Figure 4:**
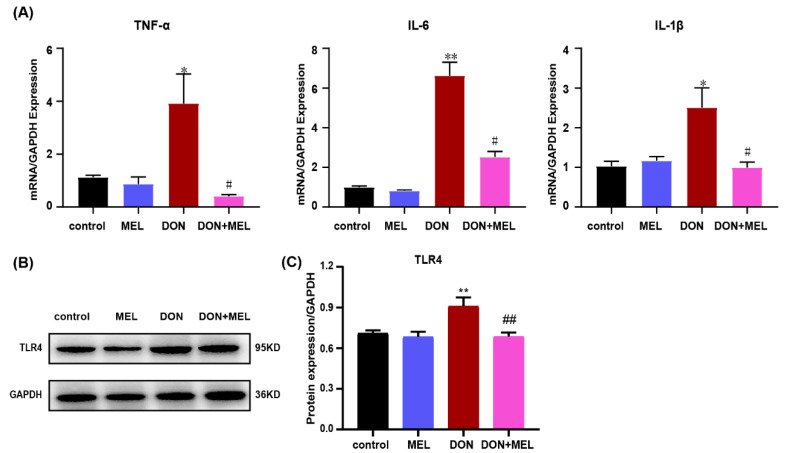
Expression of mRNAs encoding inflammatory cytokines in DON-exposed mouse ovary GCs measured using qRT-PCR. Ovary GCs were stimulated with DON, melatonin (MEL), and DON+MEL treated for 24 h. (**A**) The relative expression kevels of *Tnfa*, *Il6,* and *Il1b* were calculated by dividing their values by the *Gapdh* mRNA expression levels. All data are expressed as the means ± SEM. (**B**,**C**) TLR4 (1:1000 dilution) protein levels were examined in GCs after different treatments. GAPDH (1:5000 dilution) was used as internal control. * *p* < 0.05 and ** *p* < 0.01 compared with the control and MEL groups; # *p* < 0.05 and ## *p* < 0.01 compared with the DON group. MEL: melatonin.

DON treatment can cause an inflammatory response in many cell types [33]. To evaluate the protective effect of melatonin on the inflammatory response induced by DON treatment, the mRNA expression levels of *Tnfα* (tumor necrosis factor alpha), *Il6* (interleukin 6), and *Il1β* (interleukin 1 beta) were measured using qRT-PCR. *Tnfa*, *Il6*, and *Il1b* levels in DON-induced ovarian GCs were upregulated significantly compared with those in the control and melatonin groups, while this effect was mitigated significantly by the addition of melatonin (Figure 4A). Moreover, Toll-like receptor 4 (TLR4) signaling plays a vital role in the inflammatory response [34]; therefore, we investigated whether DON and melatonin affect TLR4 signaling using western blotting. The results indicated that melatonin inhibited the activation of TLR4 induced by DON in murine ovary GCs (*p <* 0.05, Figure 4B,C).

### 3.7. Melatonin Inhibits DON-Induced Downregulation of Reproduction Hormone Genes

**Figure 5:**
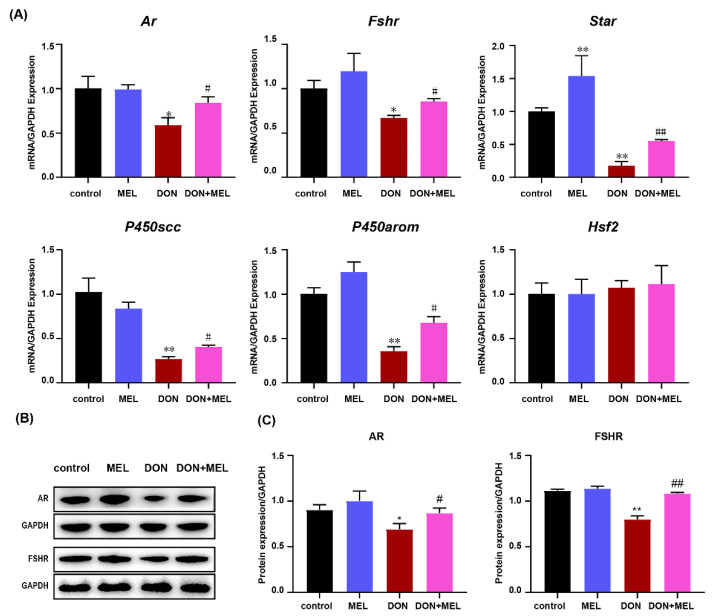
Effect of melatonin on DON-induced expression of reproductive hormone-related genes in mouse ovary GCs, measured by qRT-PCR and western blotting. Ovarian GCs were stimulated with DON, melatonin (MEL), and DON+MEL for 24 h. (**A**) The relative expression levels of *Ar*, *Fshr*, *Star*, *P450scc*, *P450arom*, and *Hsf2* were calculated by dividing their values by the *Gapdh* mRNA expression level. All data are expressed as the means ± SEM. (**B**,**C**) The protein levels of AR (1:1000 dilution) and FSHR (1:1000 dilution) in murine ovary GCs, assessed using western blot. GAPDH (1:5000 dilution) as the control. The results from each group are shown as the mean ± SD, *n* = 3. * *p* < 0.05 and ** *p* < 0.05 compared with the control and MEL groups; # *p* < 0.05 and ## *p* < 0.05 compared with the DON group. MEL: melatonin.

Considering the important physiological functions of ovary GCs, we measured the expression of reproduction hormone genes in murine ovary GCs after different treatments. The results showed that DON significantly downregulated the expression levels of *Ar* (androgen receptor), *Fshr* (follicle-stimulating hormone receptor), *Star* (steroidogenic acute regulatory protein), *P450scc* (cytochrome P450 family 11 subfamily A member 1), and *P450arom* (cytochrome P450 family 19 subfamily A member 1), while co-treatment with melatonin and DON significantly inhibited those alterations induced by DON (Figure 5A). Meanwhile, the protein levels of AR and FSHR were measured using western blotting, the results of which were consistent with the mRNA expression of levels *Ar* and *Fshr* (Figure 5B,C). These results indicated that melatonin inhibits DON-induced downregulation of reproduction hormone gene expression in ovary GCs.

### 3.8. Melatonin Reduces DON-Induced Inflammatory Response through NF-κB and MAPK Signaling Pathways

**Figure 6:**
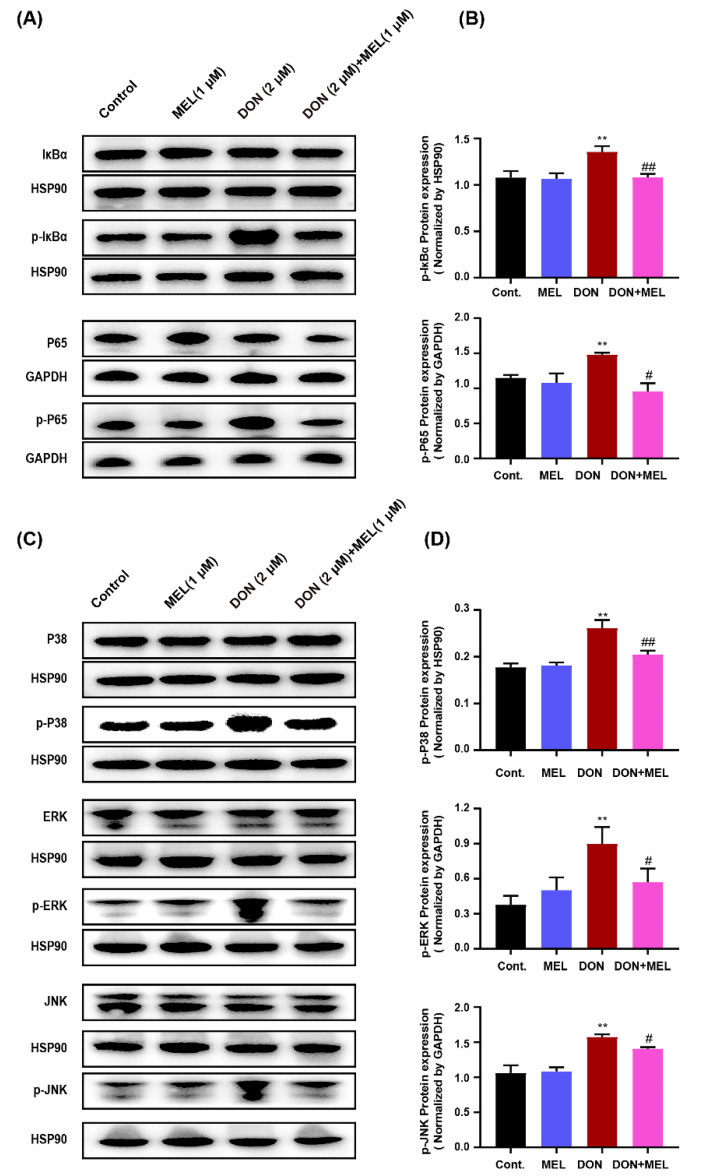
Melatonin (MEL) inhibits DON-induced NF-κB and MAPK activation in murine ovary granulosa cells. Ovarian GCs were stimulated with DON, MEL, and DON+MEL for 24 h. (**A**,**B**) IkBα (1:1000 dilution), p-IκBα (1:1000 dilution), P65 (1:1000 dilution), and p-P65 (1:1000 dilution) were examined using western blotting in GCs treated with DON, MEL, and DON+MEL for 24 h. (**C**,**D**) P38 (1:1000 dilution), p-P38 (1:1000 dilution), ERK (1:1000 dilution), p-ERK (1:1000 dilution), JNK (1:1000 dilution), and p-JNK (1:1000 dilution) were examined by western blotting in GCs. All data are shown as mean ± SD, *n* = 3. ** *p* < 0.05 compared with the control and MEL groups; # *p* < 0.05 and ## *p* < 0.05 compared with the DON group. MEL: melatonin.

To further study the anti-inflammatory effect and mechanism of melatonin on the DON-induced inflammatory response in ovary GCs, nuclear factor kappa B (NF-κB) and mitogen-activated protein kinase (MAPK) signaling pathway proteins were examined using western blotting. In the NF-κB signaling pathway, the levels of phosphorylated-p65 and phosphorylated-IκB were increased significantly in the DON-induced ovarian GCs compared with those in the control group and melatonin group. Interestingly, these changes were dramatically inhibited by the addition of melatonin (Figure 6A,B). Moreover, DON administration caused a significantly increase in the phosphorylation of extracellular regulated kinase (ERK), c-Jun N-terminal kinase (JNK), and p38 MAPKs, indicating the MAPK phosphorylation can be induced by DON. Meanwhile, melatonin treatment blocked the phosphorylation of these MAPKs (Figure 6C,D). These results suggested that melatonin might reduce the activation of NF-κB and phosphorylation of ERK, JNK, and p38 MAPKs in DON-induced ovarian GCs.

## 4. Discussion

**Figure 7:**
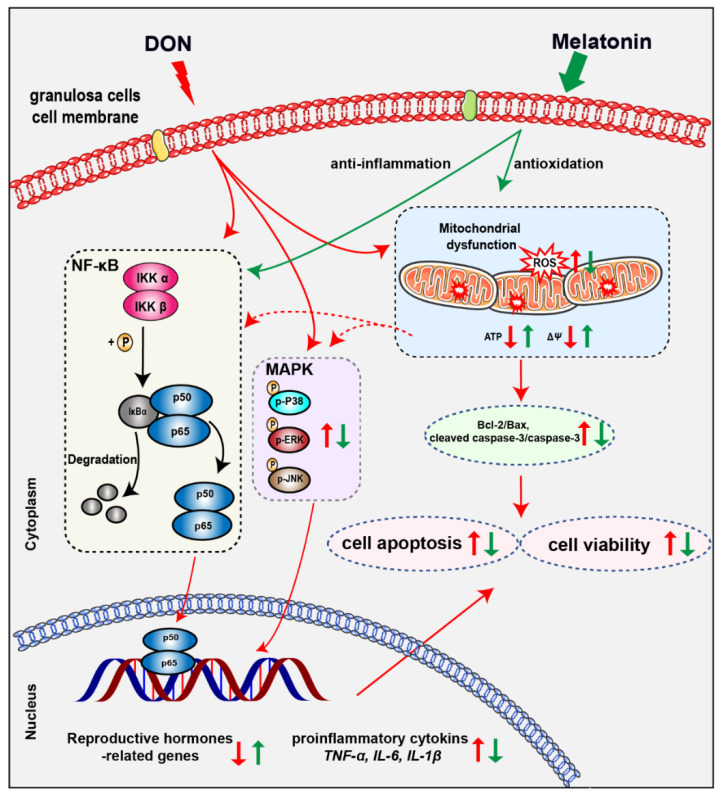
Diagram of DON exposure and melatonin administration to murine ovary granulosa cells.

In humans, the phenomena of nausea, diarrhea, and vomiting caused by moldy food are widespread. However, people are mostly unaware that DON contamination is one of causes of these hazards. DON is a type B trichothecene and is one of the most common mycotoxins found in cereals; therefore, humans are easily exposed to DON through food and water, and the level of DON could be measured by urinalysis to assess human exposure [21]. Our results indicated that cell viability decreased significantly and cell apoptosis increased significantly in murine ovary GCs exposed to DON, together with increased ROS, reduced MMP and ATP, and altered mRNA expression of inflammatory cytokine genes and reproduction hormone-genes. However, melatonin administration protected ovarian GCs from these detriments induced by DON contamination by attenuating oxidative stress-induced apoptosis and the inflammatory response. Specifically, the inhibition of NF-κB activation and the phosphorylation of MAPKs might be involved in the anti-inflammatory property of melatonin (Figure 7).

In the present study, we determined the appropriate concentration of DON that significantly inhibited cell viability and increased the apoptosis of murine ovary GCs in an in vitro experiment. The results were similar to those in DON-exposed IPEC-J2 cells [35] and DON-exposed porcine oocytes [11], and similar to the result of IPEC-J2 induced by Zearalenone [36], which is the same type of trichothecenes as DON. To further investigate the toxicity and mechanism of DON, we tested the ROS levels of ovarian GCs exposed to DON. The result showed that DON increased the ROS levels of ovarian GCs. Previous studies reported that DON could exert cytotoxicity via oxidative stress [37], and oxidative stress could induce cell apoptosis [38]. We then hypothesized that the oxidative stress induced by DON mediated the apoptosis of ovarian GCs. Melatonin is widely recognized as a natural antioxidant and free radical scavenger [11], and several articles proved the antioxidant property of melatonin [11,12,18]. Therefore, we tested the cell viability and apoptosis of ovarian GCs after co-treatment with melatonin and DON, which showed that the administration of melatonin alleviated significantly the adverse effects on cell viability and apoptosis induced by DON in murine ovary GCs. Meanwhile, the intracellular ROS level of GCs was measured using flow cytometry after co-treatment with melatonin and DON. Interestingly, our results showed that melatonin significantly decreased the ROS production induced by DON. These results confirmed our hypothesis and indicated the potential rescue effects of melatonin on DON exposed murine ovary GCs.

To further verify the protective effect and explore the potential mechanism of melatonin, mitochondrial function was analyzed in all groups. The results showed that melatonin alleviated the mitochondrial dysfunction of ovarian GCs induced by DON significantly, by mitigating the DON-induced decline of the MMP and ATP. Mitochondria are important organelles, and several studies have reported that mitochondrial function is associated with ATP generation [39], calcium homeostasis [40], ROS production [41], cytoplasmic oxidation-reduction regulation, signal transduction, and cell apoptosis. In addition, ROS are important products derived from the process of oxidative phosphorylation [42], which produces ATP as energy to cells. However, excessive ROS production triggers oxidative stress and impairs mitochondrial function [43]. DON disrupted the balance of intracellular ROS and the antioxidative capacity, leading to mitochondrial dysfunction. Moreover, the MMP is one of the important indicators that reflect mitochondrial function [13], and the decline of the MMP is a landmark event of early apoptosis. In the present study, co-treatment with DON and melatonin significantly decreased the level of intracellular ROS, and increased the MMP and intracellular ATP, suggesting that melatonin improved mitochondrial function. A previous study reported that DON inhibited mitochondrial translation, and then disrupted the MMP [44]. Furthermore, melatonin co-treatment DON significantly increased the mRNA expression levels of *Sod* and *Gshpx* compared with those in the DON group. SOD and GSH-PX are the main antioxidant enzymes, which are often used as the key index of the level of the antioxidant capacity [45]. The result indicated that GCs maintain a higher level of oxidative stress after DON exposure, while the administration of melatonin effectively reduced the level of oxidative stress. Collectively, our results indicated that the effects of melatonin on oxidative stress might be the mechanism by which it inhibits DON cytotoxicity in ovarian GCs.

Beside oxidative stress, we also tested the inflammation response induced by DON. TNF-α, IL-1β, and IL-6 are the principal proinflammatory cytokines, and their expression levels were determined in many different types of inflammatory processes, including mastitis induced by lipopolysaccharide (LPS) [34,46], the inflammatory processes of rat testis induced by cadmium [45], and mouse sertoli cells induced by LPS [47]. In the present study, we found that the administration of melatonin abrogated the upregulation of *Tnfα*, *Il1β*, and *Il6* induced by DON in murine ovary GCs. To further verify the potential inhibitory effect of melatonin on cytokine production, we assessed the changes in NF-κB and MAPK activation. Previous studies have reported that the expression levels of pro-inflammatory mediators are modulated by the NF-κB and MAPKs signaling pathways [48]. NF-κB plays a key role in cells in response to stimuli such as stress, free radicals, and bacterial and viral antigens. NF-κB exists in the form of homo-dimeric or hetero-dimeric complexes of p50 and p65 subunits bound to IκB [49]. In response to stimuli, IκB is phosphorylated by the activated IκB kinase, making the IκBα dissociate from the trimer, and then the phosphorylated IκBα is ubiquitinated and degraded. However, the dissociated NF-κB p65 rapidly enters the nucleus from the cytoplasm, binds to specific DNA sequences in the nucleus, which triggers the expression and regulation of numerous genes that are involved in the process of immune regulation, the inflammatory response, and anti-apoptosis [50]. Herein, our results showed that the administration of melatonin blocked the phosphorylation of p65 and the degradation of IκBα induced by DON significantly. These results indicated that melatonin exerts its anti-inflammatory activity by inhibiting NF-κB by blocking the phosphorylation of p65 and the degradation of IκBα. The MAPK signaling pathway plays an important role in the regulation of cell growth, differentiation, stress, inflammation, and other important physiological and pathological effects. Herein, we analyzed the protein expression of ERK1/2, p38, and JNK, which are the three main MAPKs subfamilies. The results showed that supplementation with melatonin inhibited the upregulation of the phosphorylation status of p38, ERK1/2, and JNK induced by DON. In terms of the relationship between melatonin and inflammation, melatonin plays an important role in the immune system [6]. Carrillo-Vico et al. reviewed the multiple effects of melatonin on the immune system, pointing out the pleiotropic effects of melatonin on the regulation of the immune system [8]. Meanwhile, melatonin could inhibit the proliferation, invasion, metastasis, and angiogenesis of gastrointestinal cancer cell, promote its apoptosis and cancer immunity, and exert an anti-gastrointestinal cancer effect [7]. Therefore, increasing research has focused on the application and production of melatonin because of its multiple effects. Collectively, these results demonstrated that melatonin suppressed the inflammatory response induced by DON mainly by inhibiting NF-κB and MAPKs activation. Taking our results together with those of previous studies, we determined that melatonin could inhibit the pro-inflammatory cytokine production induced by DON by preventing the activation of NF-kB and MAPKs.

Our study demonstrated that melatonin ameliorates the toxicity induced by DON in murine ovary GCs. However, regarding the actual application of melatonin to treat diseases caused by mycotoxins, many aspects still need to be optimized. For example, given that melatonin taken orally tends to show poor absolute bioavailability, an alternative method of administration may be considered. The dosage of melatonin also needs to be optimized. Furthermore, the absorption, metabolism, and side-effects of melatonin may also need to be examined to optimize its effect. Overall, our results demonstrated that melatonin ameliorates the toxicity induced by DON at the cell level in vitro, which would provide a theoretical basis to use melatonin as a drug to eliminate mycotoxin contamination.

## 5. Conclusions

In summary, these results indicate that DON exposure caused oxidative stress, inflammation-mediated apoptosis, and altered mRNA expression of reproduction hormone genes. The administration of melatonin significantly protects ovary GCs from the toxicity of DON via its anti-oxidative and anti-inflammatory capacities. Meanwhile, some unknown pathways possibly involved in melatonin ameliorate the toxicity induced by deoxynivalenol in murine ovary GCs. Combined with the protect effect of melatonin on oocytes upon DON exposure [11], melatonin can be regarded as a potential agent for prophylaxis and treatment of diseases induced by DON contamination.

## Data Availability

The data and materials supporting the conclusions are included within the article and its Supplementary files.

## Supplementary Materials

The following are available online at https://www.mdpi.com/article/10.3390/antiox10071045/s1, Table S1: Oligonucleotide sequences and size of primers. Figure S1: The morphology of murine ovary GCs treated with DON, Melatonin and DON + Melatonin.

## Abbreviations

DON: deoxynivalenol; MEL: melatonin; *Ar*: androgen receptor; *Fshr*: follicle-stimulating hormone receptor; *Star*: steroidogenic acute regulatory protein; *P450scc*: cytochrome P450 family 11 subfamily A member 1; *P450arom:* cytochrome P450 family 19 subfamily A member 1; *Hsf2*: heat shock factor 2; MMP: Mitochondrial Membrane Potential; ERK: extracellular regulated kinase; JNK: c-Jun N-terminal kinase; PCNA: proliferating cell nuclear antigen; CDK1: cyclin-dependent kinases 1; CCND2: cyclin D2; NF-κB: nuclear factor kappa B; MAPK: mitogen-activated protein kinase.

## References

[1] Li R., Albertini D.F. (2013). The road to maturation: Somatic cell interaction and self-organization of the mammalian oocyte. Nat. Rev. Mol. Cell Biol..

[2] Robinson J.W., Zhang M., Shuhaibar L.C., Norris R.P., Geerts A., Wunder F., Eppig J.J., Potter L.R., Jaffe L.A. (2012). Luteinizing hormone reduces the activity of the NPR2 guanylyl cyclase in mouse ovarian follicles, contributing to the cyclic GMP decrease that promotes resumption of meiosis in oocytes. Dev. Biol..

[3] Manabe N., Goto Y., Matsuda-Minehata F., Inoue N., Maeda A., Sakamaki K., Miyano T. (2004). Regulation Mechanism of Selective Atresia in Porcine Follicles: Regulation of Granulosa Cell Apoptosis during Atresia. J. Reprod. Dev..

[4] Cai L., Sun A., Li H., Tsinkgou A., Yu J., Ying S., Chen Z., Shi Z. (2015). Molecular mechanisms of enhancing porcine granulosa cell proliferation and function by treatment in vitro with anti-inhibin alpha subunit antibody. Reprod Biol Endocrinol..

[5] Gore-Langton R.E. (1990). Follicle-stimulating hormone and estradiol regulate antrum-like reorganization of granulosa cells in rat preantral follicle cultures. Biol. Reprod..

[6] Reiter R.J., Mayo J.C., Tan D.X., Sainz R.M., Alatorre-Jimenez M., Qin L. (2016). Melatonin as an antioxidant: Under promises but over delivers. J. Pineal Res..

[7] Xin Z., Jiang S., Jiang P., Yan X., Fan C., Di S., Wu G., Yang Y., Reiter R.J., Ji G. (2015). Melatonin as a treatment for gastrointestinal cancer: A review. J. Pineal Res..

[8] Carrillo-Vico A., Guerrero J.M., Lardone P.J., Reiter R.J. (2005). A Review of the Multiple Actions of Melatonin on the Immune System. Endocrine.

[9] Gonzalez-Arto M., Hamilton T.R.D.S., Gallego M., Gaspar-Torrubia E., Aguilar D., Serrano-Blesa E., Abecia J., Pe R.M.P., Blanco M.T.M., Pérez J. (2016). Evidence of melatonin synthesis in the ram reproductive tract. Andrology.

[10] Reiter R.J., Tamura H., Tan D.X., Xu X.Y. (2014). Melatonin and the circadian system: Contributions to successful female reproduction. Fertil. Steril..

[11] Lan M., Han J., Pan M.H., Wan X., Pan Z.N., Sun S.C. (2018). Melatonin protects against defects induced by deoxynivalenol during mouse oocyte maturation. J. Pineal Res..

[12] Suwannakot K., Sritawan N., Prajit R., Aranarochana A., Sirichoat A., Pannangrong W., Wigmore P., Welbat J. (2021). Melatonin Protects against the Side-Effects of 5-Fluorouracil on Hippocampal Neurogenesis and Ameliorates Antioxidant Activity in an Adult Rat Hippocampus and Prefrontal Cortex. Antioxidants.

[13] Zou H., Chen B., Ding D., Gao M., Chen D., Liu Y., Hao Y., Zou W., Ji D., Zhou P. (2020). Melatonin promotes the development of immature oocytes from the COH cycle into healthy offspring by protecting mitochondrial function. J. Pineal Res..

[14] Li Y., Zhang Z., He C., Zhu K., Xu Z., Ma T., Tao J., Liu G. (2015). Melatonin protects porcine oocyte in vitro maturation from heat stress. J. Pineal Res..

[15] Liu Y., Yang Y., Li W., Ao H., Zhang Y., Zhou R., Li K. (2019). Effects of melatonin on the synthesis of estradiol and gene expression in pig granulosa cells. J. Pineal Res..

[16] He C., Wang J., Li Y., Zhu K., Xu Z., Song Y., Liu G. (2015). Melatonin-related genes expressed in the mouse uterus during early gestation promote embryo implantation. J. Pineal Res..

[17] Tian X., Wang F., He C., Zhang L., Tan D., Reiter R.J., Xu J., Ji P., Liu G. (2014). Beneficial effects of melatonin on bovine oocytes maturation: A mechanistic approach. J. Pineal Res..

[18] Zhang Y., Wang T., Lan M., Zang X.-W., Li Y.-L., Cui X.-S., Kim N.-H., Sun S.-C. (2018). Melatonin protects oocytes from MEHP exposure-induced meiosis defects in porcine. Biol. Reprod..

[19] Urbanek K.A., Habrowska-Gorczynska D.E., Kowalska K., Stanczyk A., Dominska K., Piastowska-Ciesielska A.W. (2018). Deoxynivalenol as potential modulator of human steroidogenesis. J. Appl. Toxicol..

[20] Han J., Wang Q.-C., Zhu C.-C., Liu J., Zhang Y., Cui X.-S., Kim N.-H., Sun S.-C. (2016). Deoxynivalenol exposure induces autophagy/apoptosis and epigenetic modification changes during porcine oocyte maturation. Toxicol. Appl. Pharmacol..

[21] Wang Z., Wu Q., Kuca K., Dohnal V., Tian Z. (2014). Deoxynivalenol: Signaling pathways and human exposure risk assessment--an update. Arch. Toxicol..

[22] Wu Q., Lohrey L., Cramer B., Yuan Z., Humpf H.U. (2011). Impact of physicochemical parameters on the decomposition of deoxynivalenol during extrusion cooking of wheat grits. J. Agric. Food Chem..

[23] Medvedova M., Kolesarova A., Capcarova M., Labuda R., Sirotkin A.V., Kovacik J., Bulla J. (2011). The effect of deoxynivalenol on the secretion activity, proliferation and apoptosis of porcine ovarian granulosa cells in vitro. J. Environ. Sci. Health Part B..

[24] Wang H., Zong Q., Wang S., Zhao C., Wu S., Bao W. (2019). Genome-Wide DNA Methylome and Transcriptome Analysis of Porcine Intestinal Epithelial Cells upon Deoxynivalenol Exposure. J. Agric. Food Chem..

[25] Knutsen H.K., Alexander J., Barregard L., Bignami M., Bruschweiler B., Ceccatelli S., Cottrill B., Dinovi M., Grasl-Kraupp B., EFSA Panel on Contaminants in the Food Chain (CONTAM) (2017). Risks to Human and Animal Health Related to the Presence of Deoxynivalenol and Its Acetylated and Modified Forms in Food and Feed.

[26] Borutova R., Faix S., Placha I., Gresakova L., Cobanova K., Leng L. (2008). Effects of deoxynivalenol and zearalenone on oxidative stress and blood phagocytic activity in broilers. Arch. Anim. Nutr..

[27] Alizadeh A., Braber S., Akbari P., Garssen J., Fink-Gremmels J. (2015). Deoxynivalenol Impairs Weight Gain and Affects Markers of Gut Health after Low-Dose, Short-Term Exposure of Growing Pigs. Toxins.

[28] Guerrero-Netro H.M., Chorfi Y., Price C.A. (2015). Effects of the mycotoxin deoxynivalenol on steroidogenesis and apoptosis in granulosa cells. Reproduction.

[29] Alm H., Greising T., Brussow K.-P., Torner H., Tiemann U. (2002). The influence of the mycotoxins deoxynivalenol and zearalenol on in vitro maturation of pig oocytes and in vitro culture of pig zygotes. Toxicol. Vitro.

[30] Minervinia F., Dell’Aquilab M.E., Maritatob F., Minoiab P., Viscontia A. (2001). Toxic effects of the mycotoxin zearalenone and its derivatives on in vitro maturation of bovine oocytes and 17β-estradiol levels in mural granulosa cell cultures. Toxicol. Vitro.

[31] Alm H., Brüssow K.-P., Torner H., Vanselow J., Tomek W., Dänicke S., Tiemann U. (2006). Influence of Fusarium-toxin contaminated feed on initial quality and meiotic competence of gilt oocytes. Reprod. Toxicol..

[32] Livak K.J., Schmittgen T.D. (2001). Analysis of relative gene expression data using real-time quantitative PCR and the 2(-Delta Delta C(T)) Method. Methods.

[33] Waskiewicz A., Beszterda M., Kostecki M., Zielonka L., Golinski P., Gajecki M. (2014). Deoxynivalenol in the gastrointestinal tract of immature gilts under per os toxin application. Toxins.

[34] Wei W., Dejie L., Xiaojing S., Tiancheng W., Yongguo C., Zhengtao Y., Naisheng Z. (2015). Magnolol inhibits the inflammatory response in mouse mammary epithelial cells and a mouse mastitis model. Inflammation.

[35] Kang R., Li R., Dai P., Li Z., Li Y., Li C. (2019). Deoxynivalenol induced apoptosis and inflammation of IPEC-J2 cells by promoting ROS production. Environ. Pollut..

[36] Fan W., Shen T., Ding Q., Lv Y., Li L., Huang K., Yan L., Song S. (2017). Zearalenone induces ROS-mediated mitochondrial damage in porcine IPEC-J2 cells. J. Biochem. Mol. Toxicol..

[37] Mishra S., Dwivedi P.D., Pandey H.P., Das M. (2014). Role of oxidative stress in Deoxynivalenol induced toxicity. Food Chem. Toxicol..

[38] Wang X., Xu W., Fan M., Meng T., Chen X., Jiang Y., Zhu D., Hu W., Gong J., Feng S. (2016). Deoxynivalenol induces apoptosis in PC12 cells via the mitochondrial pathway. Environ. Toxicol. Pharmacol..

[39] Andersson S.G., Karlberg O., Canback B., Kurland C.G. (2003). On the origin of mitochondria: A genomics perspective. Phil. Trans. R Soc. Lond. B.

[40] Marchi S., Patergnani S., Missiroli S., Morciano G., Rimessi A., Wieckowski M., Giorgi C., Pinton P. (2018). Mitochondrial and endoplasmic reticulum calcium homeostasis and cell death. Cell Calcium..

[41] Pivovarova N.B., Andrews S.B. (2010). Calcium-dependent mitochondrial function and dysfunction in neurons. FEBS J..

[42] Palmeira C.M., Teodoro J.S., Amorim J.A., Steegborn C., Sinclair D.A., Rolo A.P. (2019). Mitohormesis and metabolic health: The interplay between ROS, cAMP and sirtuins. Free. Radic. Biol. Med..

[43] He L., He T., Farrar S., Ji L., Liu T., Ma X. (2017). Antioxidants Maintain Cellular Redox Homeostasis by Elimination of Reactive Oxygen Species. Cell. Physiol. Biochem..

[44] Bin-Umer M.A., McLaughlin J.E., Basu D., McCormick S., Tumer N.E. (2011). Trichothecene mycotoxins inhibit mitochondrial translation--implication for the mechanism of toxicity. Toxins.

[45] Liu X.R., Wang Y.Y., Fan H.R., Wu C.J., Kumar A., Yang L.G. (2016). Preventive effects of beta-cryptoxanthin against cadmium-induced oxidative stress in the rat testis. Asian J. Androl..

[46] Li D., Fu Y., Zhang W., Su G., Liu B., Guo M., Li F., Liang D., Liu Z., Zhang X. (2013). Salidroside attenuates inflammatory responses by suppressing nuclear factor-kappaB and mitogen activated protein kinases activation in lipopolysaccharide-induced mastitis in mice. Inflamm. Res..

[47] Liu X.R., Wang Y.Y., Dan X.G., Kumar A., Ye T.Z., Yu Y.Y., Yang L.G. (2016). Anti-inflammatory potential of beta-cryptoxanthin against LPS-induced inflammation in mouse Sertoli cells. Reprod. Toxicol..

[48] Fu Y., Liu B., Zhang N., Liu Z., Liang D., Li F., Cao Y., Feng X., Zhang X., Yang Z. (2013). Magnolol inhibits lipopolysaccharide-induced inflammatory response by interfering with TLR4 mediated NF-κB and MAPKs signaling pathways. J. Ethnopharmacol..

[49] Oh Y.-C., Cho W.-K., Jeong Y.H., Im G.Y., Kim A., Hwang Y.-H., Kim T., Song K.H., Ma J.Y. (2012). A Novel Herbal Medicine KIOM-MA Exerts an Anti-Inflammatory Effect in LPS-Stimulated RAW 264.7 Macrophage Cells. Evid. Based Complement. Altern. Med..

[50] Hayden M.S., Ghosh S. (2012). NF-kappaB, the first quarter-century: Remarkable progress and outstanding questions. Genes Dev..

