# Supplementary material for: Melatonin Ameliorates the Toxicity Induced by Deoxynivalenol in Murine Ovary Granulosa Cells by Antioxidative and Anti-Inflammatory Effects
Source: Antioxidants (Basel). 2021 Jun 29;10(7):1045. doi: 10.3390/antiox10071045 (PMC8300713; doi:10.3390/antiox10071045)
Supplement: Supplementary file 1 [file antioxidants-10-01045-s001.zip › antioxidants-1230203-supplementary.pdf]

Supplementary Files

Table S1. Oligonucleotide sequences and size of primers.

No.	Gene Name	Primer pairs (5'→3')	Product size(bp)
1	*Sod*	AAAGCGGTGTGCGTGCTGAA<br>CAGGTCTCCAACATGCCTCT	246
2	*Gshpx*	CCTCAAGTACGTCCGACCTG<br>CAATGTCGTTGCGGCACACC	196
3	*Tnfa*	GTCCCCAAAGGGATGAGAAGTT<br>GTTTGCTACGACGTGGGCTACA	125
4	*Il1b*	TGTTTTCCTCCTTGCCTCTGAT<br>GAGTGCTGCCTAATGTCCCCTT	106
5	*Il6*	GAGGATACCACTCCCAACAGACC<br>AAGTGCATCATCGTTGTTCATACA	141
6	*Ar*	AAAATCCCACATCCTGCTCAA<br>GGAAAGTCCACGCTCACCA	136
7	*Fshr*	GCAAGCCCAGATTTACAAG<br>TGATTTAGAGGGACAAGCAC	120
8	*Star*	AGCTCTCTGCTTGGTTCTCAA<br>TTAGCACTTCGTCCCCGTTC	163
9	*P450scc*	GACCTATTCCGCTTTTCCT<br>AAGTCTCGCTTCTGCCTTA	281
10	*P450arom*	CCTGGCTACTGTCTGGG<br>GGCTCGGGTTGTTGTTA	271
11	*Hsf2*	TCGGAAGATTGTCCAGTTTATTG<br>AGTTGGTTCTTTGACTATGTGCTG	139
12	*Gapdh*	GGAGAGTGTTTCCTCGTCCC<br>ACTGTGCCGTTGAATTTGCC	202

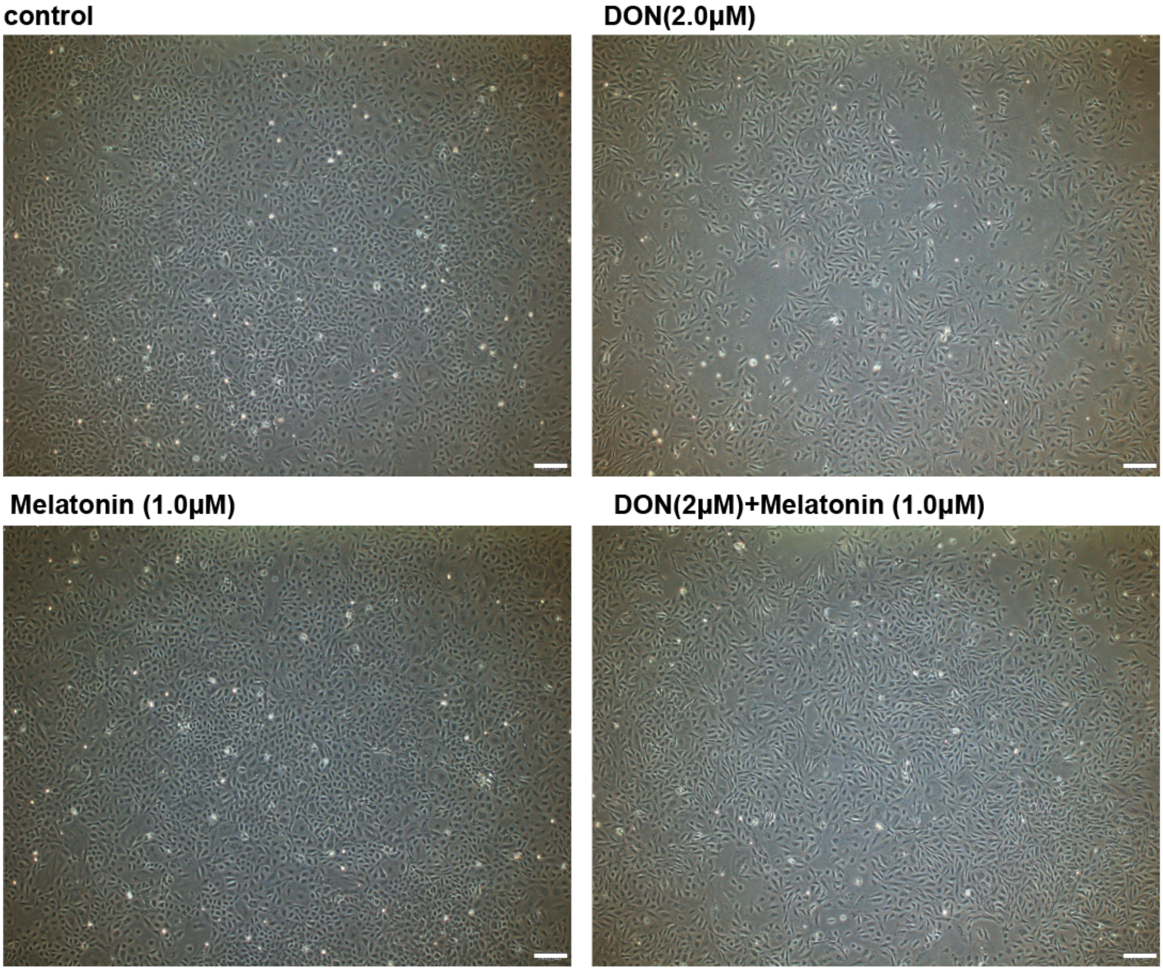

Figure S1. The morphology of murine ovary GCs treated with DON, Melatonin and DON + Melatonin. Scale bar, 200 μm.
